# Potentially Inappropriate Medication Use Among Older Adults with Cognitive Impairment and Dementia Attending Primary Care-Based Memory Clinics †

**DOI:** 10.3390/pharmacy13030082

**Published:** 2025-06-07

**Authors:** Rishabh Sharma, Linda Lee, Feng Chang, Tejal Patel

**Affiliations:** 1 School of Pharmacy, University of Waterloo, 10 Victoria St S A, Kitchener, ON N2G 1C5, Canada; r367shar@uwaterloo.ca (R.S.); feng.chang@uwaterloo.ca (F.C.); 2 CFFM MINT Memory Clinic, 25 Joseph St, Kitchener, ON N2G 4X6, Canada; lee.linda.lw@gmail.com; 3 Schlegel-UW Research Institute for Aging, 250 Laurelwood Dr, Waterloo, ON N2J 0E2, Canada; 4 Department of Family Medicine, McMaster University, 100 Main St W 5th Floor, Hamilton, ON L8P 1H6, Canada

**Keywords:** older adults, dementia, potentially inappropriate medication, memory clinic

## Abstract

Potentially inappropriate medications (PIMs) increase the risk of adverse drug reactions, hospitalizations, and worsened health outcomes in older adults, particularly those with cognitive impairment (CI) or dementia. This study was designed to compare the Beers Criteria^®^ 2023 and the Screening Tool of Older Persons’ Potentially Inappropriate Prescriptions (STOPP) Criteria 2023 to determine which identifies a higher prevalence of PIMs in older adults with CI or dementia attending primary care-based memory clinics. PIMs were identified with the use of the updated Beers Criteria^®^ 2023 and STOPP Criteria 2023, from electronic medical records of study participants from January to August 2023. The study identified PIMs and analyzed associated risk factors using bivariate logistic regression. Of 44 older adults, 47.7% (n = 21) were detected with one PIM based on Beers Criteria^®^ 2023, and 27.2% (n = 12) were identified with at least one PIM using STOPP criteria. Using the updated Beers Criteria^®^ 2023 and STOPP Criteria 2023, the study identified 50 PIMs (averaging 0.9 PIMs per participant) based on Beers Criteria^®^ and 31 PIMs (averaging 0.6 PIMs per participant) based on STOPP Criteria, respectively. Bivariate logistic regression revealed a significant association between having nine or more comorbidities and PIMs according to Beers Criteria^®^ (odds ratio (OR) = 8.4, 95% confidence interval (CIn) = 1.27–55.39, *p* = 0.027). This study highlights the high prevalence of PIMs among older adults with CI or dementia, emphasizing the need for regular medication reviews. Implementing both criteria can enhance medication management and improve patient safety in this vulnerable population.

## 1. Introduction

Dementia is a progressive syndrome marked by a decline in cognitive functions, such as thinking, remembering, and reasoning, that interferes with daily activities [1]. As people age, their risk of dementia rises, and, as such, the global aging population has seen a rising prevalence of neurodegenerative diseases, particularly dementia [2]. Approximately 402,000 older adults in Canada are affected by dementia, with 76,000 new cases yearly [3,4]. Research projections estimate that by 2030, about one million Canadians will suffer from dementia, rising to over 1.7 million by 2050 [5]. Individuals with dementia experience higher mortality rates and face numerous health challenges [6]. Economically, dementia imposes substantial costs on healthcare systems due to medical care and lost productivity. In 2016, the annual cost of dementia in Canada was CAD 10.4 billion, and it is projected to increase by 60% by 2031, reaching CAD 16.6 billion [7].

Older adults with dementia frequently have multiple comorbid conditions, including diabetes, coronary artery disease, stroke, hypertension, and heart failure [8]. On average, they experience 4.6 chronic illnesses alongside dementia, compared to an average of two comorbidities in those without dementia. This population also faces higher prevalence rates of geriatric syndromes like delirium, falls, and incontinence [9]. The presence of these comorbidities raises the likelihood of being admitted to the hospital, prolongs hospital stays, and raises the cost of healthcare [10]. Managing these conditions typically requires multiple medications to address each health concern effectively. Older adults with dementia often experience polypharmacy [8]. Over half of these individuals are prescribed five or more medications daily, with an average of eight drugs compared to three for those without dementia [11]. Subsequently, this group has a greater susceptibility to the use of potentially inappropriate medications (PIM) and resulting adverse effects and drug-related problems (DRPs) [12,13].

PIMs are medications whose adverse risks outweigh their benefits, particularly when safer alternatives are available [13]. PIM usage in older persons is associated with a greater risk of falls, extended hospital stays, adverse events, DRPs, and overall healthcare expenses [14,15,16]. Studies have indicated a high prevalence of PIM usage in older adults with cognitive impairment (CI) or dementia, ranging from 10% to 64% across various settings and countries [12,17,18]. A systematic review by Patel et al. found that 15% to 46.8% of older adults with CI or dementia in the community used PIMs, with anticholinergics and benzodiazepines being the most common [13]. Roux et al. used the Quebec Integrated Chronic Disease Surveillance System to study older persons who lived in communities in Quebec, finding that 25% had used at least one PIM continuously for a year. Factors associated with persistent PIM use included increasing age, male, gender, polypharmacy, and chronic illnesses [19].

To evaluate the prevalence and risk variables linked to PIMs in older adults, researchers have developed a variety of implicit and explicit assessments [20,21]. The use of PIMs in older adults with dementia or CI has not been well studied in Canada, which highlights the need for more study to fully comprehend the use, risk factors, and effects of PIMs in this group within the Canadian healthcare system. Examining the prevalence of PIM use is crucial for informing medication reviews and the overall wellbeing of these individuals. Recent revisions to widely used criteria, such as the Beers Criteria^®^ 2023 and the Screening Tool of Older Persons’ Potentially Inappropriate Prescriptions (STOPP) Criteria 2023, have been developed considering the increasing frequency of PIMs in older adults [13]. This study aims to compare the Beers Criteria^®^ 2023 and the STOPP Criteria 2023 in memory clinics, aiming to determine which of these criteria identifies a higher prevalence of PIMs in older adults with CI or dementia attending primary care-based memory clinics.

## 2. Materials and Methods

### 2.1. Study Design and Study Setting

This cross-sectional study was conducted at a Multispecialty Interprofessional Team-based (MINT) memory clinic in Kitchener-Waterloo. MINT memory clinics offer comprehensive assessment, diagnosis, and treatment for memory and CI, including Alzheimer’s disease and other forms of dementia [22,23]. These clinics are staffed by an interdisciplinary team of healthcare professionals specializing in the care of patients with memory-related conditions [22,23].

### 2.2. Study Population

This study included older adults (age ≥ 65) of both sex who were diagnosed with dementia or CI, receiving care at a MINT memory clinic, taking one or more medications (prescription or over the counter), and willing to provide consent. If the patient was unable to provide informed consent, a caregiver’s consent was sought. This occurred only in instances where the caregiver was actively involved in the patient’s day-to-day activities and could assist in making the decision. Exclusion criteria were individuals unwilling to give informed consent, those taking only natural health products (NHPs), and those with CI as a part of normal aging.

### 2.3. Study Procedures

#### 2.3.1. Screening and Recruitment

Between January and August of 2023, participants with dementia or CI were identified and selected at a MINT memory clinic based on specific inclusion and exclusion criteria and offered participation in the study. Consent was obtained from eligible participants or their caregivers if the participant could not consent.

#### 2.3.2. Data Collection

Data was gathered from electronic medical records for every study participant using a standardized form. Sex, age, marital status, social history (drinking and smoking), current health issues, allergies, recent falls, prescription medicines (including dosage, route, regimen, instructions, indication, and start date), and serum creatinine values were among the information gathered. Both prescription and over-the-counter medications were categorized using the Anatomical Therapeutic Chemical (ATC) system [24]. Participant serum creatinine lab values were also extracted from the patient medical records. The Cockcroft–Gault equation was used to determine the creatinine clearance (CrCl) for study participants based on their serum creatinine level [25].

#### 2.3.3. Evaluation of Potentially Inappropriate Medication Use

The 2023 American Geriatric Society (AGS) Beers Criteria^®^ and the STOPP Criteria 2023 were used to identify PIMs and were categorized into the following groups: 1. Independent of diagnosis (medications that should be avoided in older adults aged 65 or more, irrespective of their diagnosis due to their high potential for adverse effects). 2. Dependent on diagnosis (medications that should be avoided among older adults based on their specific health conditions or diagnosis). 3. Used with caution (medications that require careful consideration or monitoring due to an increased risk of adverse events). 4. Potentially clinically important drug–drug interactions. 5. Based on the patient’s kidney function (medications that should be avoided or their dosage should be reduced based on patient kidney function [26,27]). A researcher (RS) analyzed and evaluated the prescription drugs for each participant in this study.

### 2.4. Statistical Analysis

Statistical analysis was performed using the IBM Statistical Package for Social Science Statistics for Windows, Version 24.0 (SPSS Inc., Chicago, IL, USA) and STATA version Stata/SE 15.0 for Windows (Cor, 2017) [28,29]. Percentages represented categorical variables, while means ± standard deviations (SDs) or medians ± interquartile ranges (IQRs) described continuous variables, based on data normality. Bivariate logistic regression analysis identified factors associated with PIM use, including study participant demographics (age and sex) and clinical characteristics (number of comorbidities and prescribed medications). Results are presented as odds ratios (ORs) with 95% confidence intervals (CIn), and a *p*-value of less than 0.05 indicated statistical significance.

## 3. Results

Over a nine-month period, 44 participants were included in the study, consisting of 20 females (45.5%) and 24 males (54.5%), with an average age of 80.2 years (standard deviation (SD) +/−6.2). One-fourth (11 participants) were aged 85 or older, while 60% (26 participants) were aged between 75 and 84 years. The participants were assessed using both the Beers Criteria^®^ and STOPP Criteria. Detailed baseline and clinical characteristics are in Table 1. Among the participants, 36.4% (sixteen participants) had mild cognitive impairment (MCI), 20.5% (nine participants) had mixed dementia, and 11.4% (five participants) had vascular CI. Most participants (61.4%, 28 participants) had six or more comorbidities, with a mean of 6.7 (SD 3.4). The most prevalent comorbidities were hypertension (63.6%, 28 participants), hyperlipidemia (31.8%, 14 participants), chronic kidney disease (27.2%, 12 participants), and obstructive sleep apnea (25%, 11 participants). Additionally, 52.3% (23 participants) had a history of falls, detailed in Table 1.

### 3.1. Medication Use

Among the 44 participants in the study, a total of 375 medications were prescribed (Table 1). The median number of daily medications was 7.5, with a range of 1 to 21 per person. Nearly half (47.7%, 21 participants) were taking 5–9 medications daily (polypharmacy), while 38.6% (17 participants) were on 10 or more medications daily (hyper polypharmacy). The most prescribed medication categories were those for the nervous system (81.8%, 36 participants), cardiovascular system (81.8%, 36 participants), and blood and blood-forming organs (77.3%, 34 participants).

### 3.2. PIMs

In this study, 47.7% (21 participants) used at least one PIM based on Beers Criteria^®^, and 27.2% (12 participants) based on STOPP Criteria, as shown in Table 2. The Beers Criteria^®^ identified 50 PIMs, averaging 0.9 PIMs per participant, while the STOPP Criteria identified 31 PIMs, averaging 0.6 PIMs per participant.

#### 3.2.1. PIMs Identified Using Beers Criteria^®^ 2023

Nearly 50% (25) of the identified PIMs fell into the diagnosis-dependent category, with the history of falls or fractures being the most dominant. The diagnosis-independent category accounted for 32% (16) of the PIMs, predominantly involving central nervous system drugs. Among these, atypical antipsychotics such as risperidone were identified as PIMs in three participants. Additionally, seven PIMs were identified in the potentially clinically important drug–drug interaction (DDI) category, and only one PIM each was identified in the drugs used with caution and according to kidney function categories, respectively (see Supplementary Table S1)

#### 3.2.2. PIMs Identified Using STOPP Criteria 2023

Of these thirty-one PIMs, 61.3% (nineteen) were in the diagnosis-dependent category, and 25.9% (eight) were in the diagnosis-independent category (see Supplementary Table S2). In the diagnosis-dependent category, the most common PIMs were those that increase fall risk in older adults, which matched the drugs listed under history of falls and fractures in the Beers Criteria^®^. However, PIMs identified for dementia or CI in the Beers Criteria^®^ were not included in the STOPP Criteria. On the other hand, PIMs related to bradycardia, insomnia, and constipation in the STOPP Criteria were absent from the Beers Criteria^®^. In the diagnosis-independent category, apixaban was identified as a PIM in two participants using the STOPP Criteria. Additionally, there were three PIMs identified from drug–drug interactions and only one PIM from the category based on estimated Glomerular Filtration Rate (eGFR) in the STOPP Criteria.

### 3.3. Factors Associated with PIMs as per Beer’s Criteria^®^ and STOPP Criteria

The findings of the binary logistic regression test are shown in Table 3, which examined the association between PIMs determined by Beers and STOPP Criteria and independent risk factors such as age, sex, number of comorbidities, number of medications used daily, nervous system drugs, and recent history of falls. A significant association was found between age > 80 years and the occurrence of PIMs according to STOPP Criteria. For those over 80, the odds of having a PIM based on Beers Criteria^®^ were 2.52 times higher, but this difference was not statistically significant. Using STOPP Criteria, the odds were 4.38 times higher, with marginal statistical significance (OR = 4.38, 95% CIn = 0.99–19.35, P = 0.051). A significant association was observed between having nine or more comorbidities and PIMs according to Beers Criteria^®^ (OR = 8.4, 95% CIn = 1.27–55.39, P = 0.027), though no significant association was found with STOPP Criteria. The number of drugs used daily (continuous variable) (OR = 1.14, 95% CIn = 0.99–1.32, P = 0.064) and a recent history of falls (OR = 3.11, 95% CIn = 0.90–10.69, P = 0.072) were two additional marginally significant relationships found with Beers Criteria^®^.

## 4. Discussion

This study was conducted with the primary aim of comparing the Beers Criteria^®^ 2023 and the STOPP Criteria 2023 in a memory clinic setting. We sought to determine which of these criteria might be more useful for ongoing and future use in memory clinics, where medication management for this population is critical.

The research investigated PIM use in older adults with dementia or CI, comparing the Beers Criteria^®^ 2023 and the STOPP Criteria 2023. Using the Beers Criteria^®^, 47.7% (n = 21) of participants were identified with at least one PIM, while 27.2% (n = 12) were identified using STOPP Criteria. The prevalence of PIMs identified with the Beers Criteria^®^ was consistent with previous studies, but the prevalence reported using STOPP Criteria was lower [30]. A 2016 systematic review found that PIMs identified by STOPP Criteria ranged from 32.4% to 66.8% [31]. Variability in PIM prevalence can be influenced by factors such as the study population, healthcare setting in which the research was conducted, and the criteria used (Beers Criteria^®^ from 2012, 2015, 2019, and 2023 or STOPP Criteria 2015 and 2023) [32].

A meta-analysis including 11 studies found that 38% of older adults with dementia or CI attending memory clinics use PIMs, with anticholinergics, benzodiazepines, and sedatives being most common. Polypharmacy prevalence was 60%, and hyper-polypharmacy was 17.6%, highlighting significant variability due to differing PIM definitions and tools [33]. In their study involving community-dwelling participants with MCI or dementia attending nine memory clinics, Cross et al. identified PIMs using Beers 2012 or STOPP 2015 Criteria [34]. They defined a significant anticholinergic burden (ACB) as a score of ≥3 on the ACB scale, finding at least one PIM in 21.4% (206/967) of participants, lower than the prevalence in our study (47.7% with Beers Criteria^®^ and 27.2% with STOPP Criteria). Updated versions of the Beers and STOPP Criteria may alter the list of identified PIMs. Additionally, differences in study sample characteristics, such as demographics, health conditions, medications used, and healthcare settings, could affect the prevalence rates of identified PIMs. The most identified PIMs included anticholinergic drugs, benzodiazepines, non-benzodiazepines, and selective serotonin reuptake inhibitor (SSRIs), which align with the PIMs identified in our study [34].

Given the challenges of limited resources and time in clinical practice, it is necessary to compare tools to determine which one enables a greater detection of PIM use in our population, as the use of both tools is impractical to operationalize in a resource-limited environment. Understanding which tool provides more information in this setting could improve clinical decision-making, particularly when addressing safety and appropriateness in older adults with dementia or CI. Highlighting the differences between the Beers Criteria^®^ and STOPP Criteria is essential for evaluating medication appropriateness in older adults, including those with dementia. The use of these criteria in clinical practice may be driven by comprehensiveness and practicality of use. These distinctions assist healthcare providers in making informed decisions about medication management. Key differences in the independent-of-diagnosis category include: The Beers Criteria^®^ state that danazol should not be used in older adults unless it is warranted for clinically manifested hypogonadism [26]. Negative effects on lipid profiles and elevated blood pressure are among the cardiovascular adverse effects linked to danazol, which are particularly concerning for older adults who already have a higher risk of cardiovascular issues [35]. Sulfonylureas, like gliclazide, are likewise deemed unsuitable for use in older adultsby the Beers Criteria^®^. Sulfonylureas, compared to other medications, carry a higher risk of hypoglycemia, cardiovascular events, and overall mortality, potentially increasing the risk of cardiovascular death and ischemic stroke [36]. On the other hand, gliclazide and danazol usage is not deemed unsuitable for older adults according to the STOPP Criteria [36]. These variations show how each set of criteria has certain factors to take into account according to its focus, geographical origins, and medical problems.

It is noteworthy that, in comparison to the STOPP Criteria, the Beers Criteria^®^ designate a greater number of medicines as unsuitable in the independent-of-diagnosis group. Notably, according to Beers Criteria^®^, seven drugs have been shown to be unsuitable for older adults with dementia and/or CI. However, under the STOPP Criteria’s “independent-of-diagnosis” category, no drug was found to be unsuitable for this demographic. This variation suggests that older adults with dementia and CI may benefit from the more condition-specific and tailored considerations of the Beers Criteria^®^. The STOPP Criteria include more diagnosis categories than the Beers Criteria^®^, indicating a broader scope in addressing DRPs in older adults. This comprehensive approach allows the STOPP Criteria to cover a wider range of medical conditions and considerations that may affect medication appropriateness [27]. For instance, the STOPP Criteria consider timolol inappropriate for older adults with bradycardia, a condition not addressed by the Beers Criteria^®^. Additionally, the STOPP Criteria include categories such as insomnia and constipation, which are absent in the Beers Criteria^®^, further demonstrating the extended coverage of the STOPP Criteria [37]. There is significant variability in the drug–drug interactions (DDIs) identified by both criteria, with no common DDIs between them. The Beers Criteria^®^ identify more DDIs than the STOPP Criteria, including any combination of three or more central nervous system (CNS) active drugs. Four of the seven DDIs found by the Beers Criteria^®^ include the co-administration of opioids with medications such as gabapentin, lorazepam, and pregabalin. For older persons, the combination of opioids and other CNS-active drugs poses a serious risk of side effects such as respiratory depression, falls, excessive sedation, and CI [38]. In contrast, the STOPP Criteria highlight a potential DDI involving anti-dementia drugs, specifically donepezil (an acetylcholinesterase inhibitor) combined with bisoprolol (a beta-blocker known to cause persistent bradycardia), which can lead to cardiac conduction failure, syncope, and injury. Both criteria identified only one PIM based on kidney function and estimated Glomerular Filtration Rate (eGFR). The Beers Criteria^®^ consider gabapentin inappropriate if the participant’s creatinine clearance is less than 60 mL/min, while the STOPP criteria identify celecoxib as inappropriate for a patient with an eGFR of 40 mL/min/1.73 m^2^.

There is some agreement between the Beers Criteria^®^ and the STOPP Criteria in identifying cardiovascular and anti-thrombotic agents, such as amiodarone and rivaroxaban, as inappropriate in certain situations. This concurrence underscores the importance of these medications’ appropriateness concerns. Additionally, both criteria similarly identify drugs that are inappropriate for older adults with a history of falls. For a number of important reasons, it is imperative to consider both the Beers and STOPP Criteria when determining whether medicine is suitable, particularly for older adults and those with certain medical problems like dementia. Every collection of standards provides a distinct and thorough assessment of drug appropriateness. While there could be some similarities, one focuses on particular problems and factors that the other might overlook. For example, the Beers Criteria^®^ offer additional condition-specific advice, such as pointing out drugs that are not suitable for older adults suffering from dementia or CI.

Using both sets of criteria ensures a more thorough assessment of a patient’s medication regimen, allowing for tailored, patient-centered care [26,27]. The criteria work well together to improve the assessment of medication appropriateness overall and provide a more complete picture of a patient’s drug profile. Healthcare professionals may provide the safest and most appropriate care for older adults, especially those with dementia, by combining the Beers and STOPP Criteria into their decision-making process [26,27].

### Strength and Limitations

To our knowledge, this is the first study to utilize the 2023 American Geriatric Society Beers Criteria^®^ and the 2023 European Geriatric Society (EGS) STOPP Criteria to identify PIMs in this population. As these criteria were recently updated in 2023, our study is among the first globally to report on PIMs using these new guidelines. However, several limitations of this study must be considered. Firstly, the research was conducted exclusively with older adults receiving care at a MINT memory clinic in the Kitchener-Waterloo area, which may limit the generalizability of the findings to older patients in other settings, such as nursing homes or hospitals. Additionally, the study’s cross-sectional design only provides a snapshot of PIM use, which may not reflect changes in medication use over time. The reliance on electronic medical records for data collection may also introduce biases related to documentation accuracy and completeness.

## 5. Conclusions

In conclusion, this study provides valuable insights into the prevalence and types of PIMs among older adults with dementia or CI attending MINT memory clinics in Canada. By comparing the Beers Criteria^®^ 2023 and the STOPP Criteria 2023, it highlights significant differences in medication assessment approaches. The Beers Criteria^®^ identified a higher prevalence of PIMs and included more specific medications inappropriate for dementia patients, while the STOPP Criteria offered a broader scope, addressing a wider range of conditions. The findings underscore the importance of using both criteria for a comprehensive evaluation of medication appropriateness, ensuring tailored, patient-centered care for older adults with dementia or CI. This dual-criteria approach can enhance the safety and effectiveness of medication management in this vulnerable population.

## Figures and Tables

**Table 1:** Demographic and clinical characteristics of the study population (PIMs as per Beers Criteria^®^ and STOPP Criteria).

Characteristics	Total (N = 44) n (%)	With PIMs as per Beers Criteria^®^ (N = 21) n (%)	With PIMs as per STOPP Criteria (N = 12) n (%)
Age of the patient, mean ± SD	80.2 ± 6.2	80.7 ± 5.6	83.5 ± 4.8
65–69 years	2 (4.5)	1 (4.8)	0 (0)
70–74 years	5 (11.5)	2 (9.5)	0 (0)
75–79 years	13 (29.5)	4 (19)	2 (16.6)
80–84 years	13 (29.5)	10 (47.7)	5 (41.7)
≥85 years	11 (25)	4 (19)	5 (41.7)
Sex			
Male	24 (54.5)	11 (52.3)	4 (33.3)
Female	20 (45.5)	10 (47.7)	8 (66.7)
Marital Status			
Married	38 (86.4)	20 (95.2)	10 (83.3)
Separated	1 (2.2)	0 (0)	0 (0)
Widowed	5 (11.4)	1 (4.8)	2 (16.7)
CI/dementia			
Vascular CI	5 (11.4)	4 (19)	3 (25)
Subjective cognitive decline	4 (9.1)	1 (4.7)	0 (0)
Evolving neurocognitive disorder	3 (6.8)	2 (9.5)	2 (16.6)
MCI	16 (36.4)	8 (38)	3 (25)
Mixed dementia	9 (20.5)	4 (19)	1 (8.3)
Dementia	4 (9.1)	2 (9.5)	2 (16.6)
Probable or possible Alzheimer’s disease	3 (6.7)	0 (0)	1 (8.3)
Comorbidities			
Number of comorbidities per person, mean ± SD	6.7 ± 3.4	7.6 ± 3.0	8 ± 4.1
0	1 (2.3)	0 (0)	0 (0)
1–5	16 (36.3)	5 (23.8)	3 (25)
6–8	18 (41)	9 (42.8)	5 (41.6)
≥9	9 (20.4)	7 (33.4)	4 (33.4)
Alcohol			
Never	20 (45.5)	9 (42.8)	6 (50)
Occasional drinker	11 (25)	5 (23.8)	3 (25)
Active regular drinker	13 (29.5)	7 (33.4)	3 (25)
Smoking			
Never	24 (54.5)	14 (66.6)	7 (58.4)
Ex-smoker	17 (38.7)	6 (28.6)	5 (41.6)
Active smoker	3 (6.8)	1 (4.8)	0 (0)
Recent history of falls			
Absent	21 (47.7)	7 (33.3)	4 (33.3)
Present	23 (52.3)	14 (66.7)	8 (66.7)
Medication use			
Number of medications per person, median (IQR)	7.5 (6)	2 (1)	2.5 (1)
Medications per day			
1–4	6 (13.6)	1 (4.7)	0 (0)
5–9	21 (47.7)	10 (47.6)	6 (50)
≥10	17 (38.6)	10 (47.6)	6 (50)
**ATC classification**			
G Genito urinary system and sex hormones	16 (36.4)	8 (38.0)	3 (25)
A Alimentary tract and metabolism	32 (72.7)	18 (85.7)	11 (91.6)
B Blood and blood forming organs	34 (77.3)	17 (80.9)	11 (91.6)
C Cardiovascular system	36 (81.8)	19 (90.4)	12 (100)
S Sensory organs	4 (9.1)	3 (14.2)	2 (16.6)
N Nervous system	36 (81.8)	20 (95.2)	12 (100)
L Antineoplastic and immunomodulating agents	4 (9.1)	2 (9.5)	2 (16.6)
H Systemic hormonal preparations	7 (15.9)	4 (19)	3 (25)
R Respiratory system	9 (20.5)	7 (33.3)	3 (25)
M Musculo-skeletal system	8 (18.2)	5 (23.8)	3 (25)
D Dermatologics	7 (15.9)	2 (9.5)	1 (8.3)

SD; standard deviation, IQR; interquartile range.

**Table 2:** Distribution of patients with PIMs as per Beers Criteria^®^ and STOPP Criteria.

Characteristics	PIMs as per Beers Criteria^®^ (N = 44) n (%)	PIMs as per STOPP (N = 44) n (%)
0 PIM	23 (52.3)	32 (72.7)
1 PIM	13 (29.5)	6 (13.6)
2 PIM	2 (4.5)	3 (6.8)
3 PIM	4 (9.1)	2 (4.5)
≥4 PIM	2 (4.5)	1 (2.3)
Total number of PIMs (min–max)	50 (0–6)	31 (0–7)
Average number of PIMs per patient, mean ± SD	0.9 ± 1.3	0.6 ± 1.2
Median (IQR)	0 (1)	0 (1)

SD; standard deviation, IQR; interquartile range.

**Table 3:** Binary logistic regression.

	Beers 2023		STOPP 2023	
Characteristics	Odds Ratio (95% CIn)	*p*-Value	Odds Ratio (95% CIn)	*p*-Value
Age				
65–80 years	1 (Reference)		1 (Reference)	
>80 years	2.52 (0.74–8.52)	0.135	4.38 (0.99–19.35)	0.051
Sex				
Male	1 (Reference)		1 (Reference)	
Female	1.18 (0.36–3.87)	0.783	3.33 (0.82–13.48)	0.091
Number of comorbidities				
1–5	1 (Reference)		1 (Reference)	
6–8	2.4 (0.39–9.67)	0.218	1.79 (0.35–9.05)	0.479
≥ 9	8.4 (1.27–55.39)	**0.027**	3.73 (0.60–22.85)	0.154
Number of medications per day *	1.14 (0.99–1.32)	0.064	1.09 (0.94–1.26)	0.214
Recent history of falls				
Absent	1 (Reference)		1 (Reference)	
Present	3.11 (0.90–10.69)	0.072	2.26 (0.56–9.06)	0.247

* Continuous variable. *p*-value < 0.05 considered statistically significant.

## Data Availability

The original contributions presented in this study are included in the article/Supplementary Material. Further inquiries can be directed to the corresponding author(s).

## Supplementary Materials

The following supporting information can be downloaded at: https://www.mdpi.com/article/10.3390/pharmacy13030082/s1, Table S1: PIM as per Beers criteria; Table S2: PIM as per STOPP Criteria.
