# Supplementary material for: Potentially Inappropriate Medication Use Among Older Adults with Cognitive Impairment and Dementia Attending Primary Care-Based Memory Clinics
Source: Pharmacy (Basel). 2025 Jun 7;13(3):82. doi: 10.3390/pharmacy13030082 (PMC12196525; doi:10.3390/pharmacy13030082)
Supplement: Supplementary file 1 [file pharmacy-13-00082-s001.zip › pharmacy-3633759-supplementary.pdf]

Supplementary Table

Table S1: PIM as per Beers criteria

**Domain**	**Drugs with PIMs (N= 50) n (%)**
**Independent of diagnosis**	
**Endocrine system**	
Danazol (synthetic male testosterone)- Avoid unless indicated for confirmed hypogonadism with clinical symptoms	1 (2)
Sulfonylureas (all, including short- and longer-acting)<br><br>Gliclazide	1 (2)
**Central nervous system**	
**Antidepressant**	
Doxepin>6mg/day	1 (2)
**Benzodiazepines**	
Lorazepam	1 (2)
Concomitant use of Lorazepam with opioid analgesic (Oxycodone, Tapentadol)	1 (2)
**Nonbenzodiazepine benzodiazepine receptor agonist hypnotics ("Z-drugs")**	
Zopiclone	1 (2)
**Atypical Antipsychotics**	
Risperidone	3 (6)
Quetiapine	1 (2)
**Pain medications**	
Cyclobenzaprine	1 (2)
**Skeletal muscle relaxants**	
Methocarbamol	2 (4)

**Gastrointestinal**	
Pantoprazole use for >8 weeks unless for high-risk patients	1 (2)
**Cardiovascular and Anti-thrombotic**	
Amiodarone	1 (2)
Rivaroxaban for nonvalvular atrial fibrillation (long-term treatment)	1 (2)
**Total PIM**	16 (32)
**Dependent of Diagnosis**	
**Dementia or CI**	
Doxepin >6mg/day (Antidepressant with strong Anticholinergic properties)	1 (2)
Cyclobenzaprine (Anticholinergic)	1 (2)
Lorazepam (Benzodiazepine)	1 (2)
Risperidone	1 (2)
Quetiapine	1 (2)
Darifenacin (Anticholinergic)	1 (2)
Zopiclone (Z-drugs)	1 (2)
**History of falls or fractures**	
Doxepin >6mg/day (Antidepressant with strong Anticholinergic properties)	1 (2)
Cyclobenzaprine (Skeletal muscle relaxant, Anticholinergic)	1 (2)
Lorazepam (Benzodiazepine)	1 (2)
Oxycodone (Opioid)	2 (4)
Tapentadol (Opioid)	1 (2)
Duloxetine (SNRI)	2 (4)
Sertraline (SSRI)	3 (6)
Zopiclone (Z-drugs)	1 (2)

Citalopram (SSRI)	3 (6)
Risperidone (Atypical Antipsychotics)	1 (2)
Escitalopram (SSRI)	1 (2)
Codeine (Opioid analgesic)	1 (2)
**Total PIMs**	25 (50)
**Use with caution**	
1. Duloxetine initiated (Monitor sodium levels closely when initiating or altering doses in older adults, but sodium level last checked one year back in patient)	1 (2)
**Drug-drug interaction**	
1. Opioids + Pregabalin	1 (2)
2. Tapentadol and oxycodone (Opioid) + Lorazepam (Benzodiazepines)	1 (2)
3. Cyclobenzaprine (AC) + Doxepin >6mg/day (AC)	1 (2)
4. Doxepin (TCA) + Lorazepam (Benzodiazepines) + Tapentadol/Oxycodone (Opioids) + Cyclobenzaprine (Skeletal muscle relaxant Any ≥3 of CNS-active drugs	1 (2)
5. Gabapentin + Hydromorphone (Opioid analgesic)	1 (2)
6. Gabapentin + Oxycodone (Opioid)	1 (2)
7. Gabapentin + Sertraline (SSRI) + Oxycodone (Opioids)à Any ≥3 of CNS-active drugs	1 (2)
**Total PIMs**	7 (14)
**According to kidney function**	
Gabapentin maximum recommended dose is 600 mg/day if the patient CrCl <60mL/min	1 (2)

SSRI, Selective Serotonin Reuptake Inhibitors; SNRI, Serotonin-Norepinephrine Reuptake Inhibitors; CrCl, Creatinine Clearance; CNS, Central nervous system; TCA, Tricyclic Antidepressant; AC, Anticholinergics

Table S2: PIMs as per STOPP

**Domain**	**Drugs with PIMs (N= 31) n (%)**
**Independent of Diagnosis**	

1. Lorazepam use for ≥ 4 weeks	1 (3.2)
2. Duplicate therapy (Oxycodone, Tapentadol)	1 (3.2)
3. Apixaban (Factor Xa inhibitor)	2 (6.4)
4. Dabigatran (Direct oral anticoagulant)	1 (3.2)
5. Gabapentin for non-neuropathic pain	1 (3.2)
6. Amiodarone	1 (3.2)
7. Rivaroxaban	1 (3.2)
Total PIMs	8 (25.9)
**1. Dependent of Diagnosis**	
**Bradycardia (<50/min)**	
Timolol (beta-blocker)	1 (3.2)
**Drugs that predictably increase falls risk in older adults**	
Lorazepam (Benzodiazepines)	1 (3.2)
Zopiclone (Hypnotic Z-drugs)	2 (6.4)
Oxycodone (Opioid)	2 (6.4)
Tapentadol (Opioid)	1 (3.2)
Codeine (Opioid)	1 (3.2)
Doxepin (TCA)	1 (3.2)
Sertraline (Antidepressant, SSRI)	2 (6.4)
Citalopram (SSRI)	3 (9.6)
Risperidone (Atypical Antipsychotics)	1 (3.2)
Escitalopram (Antidepressant, SSRI)	1 (3.2)
**Insomnia**	
Zopiclone use for ≥ 2 weeks	1 (3.2)

**Constipation**	
Oxycodone (Opioid)	1 (3.2)
Tapentadol (Opioid)	1 (3.2)
Total PIMs	19 (61.3)
**Drug-drug interaction**	
Spironolactone (Aldosterone antagonist) + Candesartan (Potassium conserving drugs such as ARB) without frequent serum potassium monitoring (risk of serious hyperkaliemia, > 6.0 mmol/l; serum K should be checked at least every six months) but last time Serum potassium checked more than one year	1 (3.2)
Oxycodone (Opioid) + Doxepin (TCA) + Tapentadol (Opioid) + Cyclobenzaprine (Skeletal muscle relaxant) Concomitant use of ≥ 2 antimuscarinic/anticholinergic drugs	1 (3.2)
Donepezil (Acetylcholinesterase inhibitor) + Bisoprolol (Drugs that induce persistent bradycardia (Beta-blocker) syncope, failure, injury, risk of cardiac conduction	1 (3.2)
Total PIMs	3 (9.6)
**As per eGFR level**	
Celecoxib (NSAID's, COX-2) in patient with eGFR level 40 mL/min/1.73m^2	1 (3.2)

TCA, Tricyclic Antidepressant; SSRI, Selective Serotonin Reuptake Inhibitors; SNRI, Serotonin-Norepinephrine Reuptake Inhibitors; NSAID's, Non-Steroidal Anti-Inflammatory Drugs; COX, Cyclooxygenase; eGFR, estimated Glomerular Filtration Rate
